# A Text Message Intervention for Adolescents With Depression and Their Parents or Caregivers to Overcome Cognitive Barriers to Mental Health Treatment Initiation: Focus Groups and Pilot Trial

**DOI:** 10.2196/30580

**Published:** 2021-11-09

**Authors:** Brian Suffoletto, Tina Goldstein, David Brent

**Affiliations:** 1 Department of Emergency Medicine Stanford University Palo Alto, CA United States; 2 Department of Psychology University of Pittsburgh Pittsburgh, PA United States; 3 Department of Psychiatry University of Pittsburgh Pittsburgh, PA United States

**Keywords:** adolescent, depression, help seeking, text message, intervention

## Abstract

**Background:**

Many adolescents with depression do not pursue mental health treatment following a health care provider referral. We developed a theory-based automated SMS text message intervention (Text to Connect [T2C]) that attempts to reduce cognitive barriers to the initiation of mental health care.

**Objective:**

In this two-phase study, we seek to first understand the potential of T2C and then test its engagement, usability, and potential efficacy among adolescents with depression and their parents or caregivers.

**Methods:**

In phase 1, we conducted focus groups with adolescents with depression (n=9) and their parents or caregivers (n=9) separately, and transcripts were examined to determine themes. In phase 2, we conducted an open trial of T2C comprising adolescents with depression referred to mental health care (n=43) and their parents or caregivers (n=28). We assessed usability by examining program engagement, usability ratings, and qualitative feedback at the 4-week follow-up. We also assessed potential effectiveness by examining changes in perceived barriers to treatment and mental health care initiation from baseline to 4 weeks.

**Results:**

In phase 1, we found that the themes supported the T2C approach. In phase 2, we observed high engagement with daily negative affect check-ins, high usability ratings, and decreased self-reported barriers to mental health treatment over time among adolescents. Overall, 52% (22/42) of the adolescents who completed follow-up reported that they had attended an appointment with a mental health care specialist. Of the 20 adolescents who had not attended a mental health care appointment, 5% (1/20) reported that it was scheduled for a future date, 10% (2/20) reported that the primary care site did not have the ability to help them schedule a mental health care appointment, and 15% (3/20) reported that they were no longer interested in receiving mental health care.

**Conclusions:**

The findings from this study suggest that T2C is acceptable to adolescents with depression and most parents or caregivers; it is used at high rates; and it may be helpful to reduce cognitive barriers to mental health care initiation.

## Introduction

### Background

Approximately 6% of US adolescents aged between 12 and 17 years have a diagnosis of depression; however, >20% of those diagnosed with depression have not received treatment in the past year [1]. Furthermore, studies show that, on average, 10 years elapse from the time of mental health symptom onset to receipt of treatment [2]. Among those referred to mental health care for depression or suicidality, only 18% access mental health care within 6 months [3], which may increase only to 32% with integrated mental health services [4]. If left untreated, depression can become harder to manage [5] and puts individuals at greater risk for suicide [6].

There are many documented reasons why adolescents do not seek or receive mental health treatment. Help-seeking for mental health symptoms is a multistep process beginning with an awareness of the problem, followed by an expression of the problem and the need for help to others—which for adolescents is typically a parent or caregiver–followed by a discussion and plan for help-seeking [7]. Barriers to advancement along this process for mental health treatment include lack of health literacy, perceived stigma, concerns about confidentiality, and preference for self-reliance. These barriers are amplified in the adolescent development period [8] and are difficult to overcome when both the child and parent or caregiver may have different beliefs or barriers.

Interventions aimed at improving help-seeking for mental health have shown mixed results. A systematic review from 2012 demonstrated that mental health help-seeking interventions improve attitudes but not necessarily behaviors among young people [9]. A more recent systematic review and meta-analysis of 98 mental health help-seeking interventions found that formal help-seeking behaviors improved when the intervention was delivered to adults with or at risk of mental health problems but not when delivered to adolescents [10]. Among a review of interventions targeting mental health in adolescents specifically, almost all were delivered in schools, and three-quarters were education-based [11].

Digital behavioral interventions can overcome barriers of in-person or location-based interventions [12]; however, they have also shown mixed promise for adolescent help-seeking for mental health. In 2014, a systematic review of 18 studies of web-based interventions targeting help-seeking in young people aged between 14 and 25 years (only 4 of which included adolescents) found that although the trials did not change in help-seeking behavior, quasi-experimental and cross-sectional studies reported that interventions facilitated seeking professional help for an average of 35% of users [12]. A recent scoping review of 4 studies of digital health interventions aimed at improving help-seeking behavior or access to mental health services among parents or caregivers of 2- to 12-year-olds concluded there was some evidence of improved mental health literacy but not necessarily help-seeking [13].

### Objectives

Given the current gap in evidence for help-seeking interventions for mental health care initiation among at-risk adolescents, we developed and tested an automated SMS text message intervention (Text To Connect [T2C]) designed to reduce cognitive barriers to mental health care help-seeking among adolescents with depression and their parents or caregivers. This report details the design of our intervention and presents the results from a two-phase study. In phase 1, we conducted focus groups with adolescents with depression and their parents or caregivers separately to understand the barriers to mental health treatment and the acceptability of the T2C approach. In phase 2, we conducted an open trial of T2C for adolescents with depression referred to mental health care and their parents or caregivers. We assessed usability by examining T2C program engagement as well as usability ratings and qualitative feedback at the 4-week follow-up. We assessed potential effectiveness by examining changes in perceived barriers to mental health care and mental health care initiation from baseline to 4-weeks. Findings from this study are critical to inform the further development and evaluation of digital interventions aimed at improving help-seeking and linkage to mental health care.

## Methods

### T2C Intervention Design

#### Theoretical Basis

We designed T2C to reduce cognitive barriers to seeking mental health care. T2C was informed by the Health Belief Model [14], where perceived susceptibility or severity of depression, perceived benefits to mental health treatment, and cues to action all influence the likelihood of mental health care help-seeking. The T2C design was also shaped by the belief that help-seeking is a multistep process that starts with awareness of the problem, followed by a recognition that mental health care can help and subsequently by a discussion and plan for help-seeking between child and parents or caregivers [7].

#### Technical Design

T2C is an automated computer program created and housed in the Office of Academic Computing at the University of Pittsburgh Department of Psychiatry. In brief, it is a table-driven program that uses Microsoft SQL, which batches outgoing timed messages and receives incoming messages using Twilio (Twilio Inc). Program flow, branching logic, query wording, and feedback libraries were all created before the enrollment of the first participant. Although the program was designed to function with the child-parent or -caregiver dyad, we allowed either parent or caregiver or child to enroll without the other to enhance applicability to real-world situations.

#### Onboarding

To onboard, T2C participants were prompted to text a unique keyword to initiate T2C. Once initiated, participants received introductory texts including instructions that they could drop out of the T2C program at any time by texting *Quit*. Parents or caregivers and adolescents received a series of SMS text message queries to assist in the stratification and tailoring of T2C content.

### T2C Intervention

#### Parent or Caregiver

**Figure 1:**
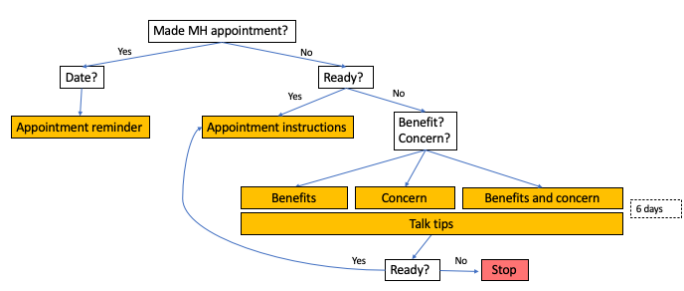
Parent or caregiver Text To Connect flow diagram. MH: mental health.

Upon enrollment (Figure 1), parents or caregivers received the message, “Have you made an appointment for your child to see one of our mental health clinicians?” If they reported that they had already made an appointment, they were sent a link to an informational page about the mental health clinicians at their site and a query, “What is the date of your appointment?” which was used to send a reminder text the day before the scheduled appointment. If the parent or caregiver reported not having made an appointment, they were asked, “Are you ready to make an appointment for your child?” If they replied affirmatively, they were provided instructions on how to set up an mental health care appointment. If they indicated not being ready, they received the messages, “Do you feel that your child could benefit from seeing a mental health clinician?” and “Do you have concerns about your child seeing a mental health clinician?” On the basis of their responses to these queries, an individual would receive psychoeducation aimed at reducing stigma, increasing perceived benefits of mental health care, or both. Psychoeducation was designed in a microlearning format [15] as daily *true* or *false* questions for 6 days (see Multimedia Appendix 1 for sample messages). To encourage conversations between parent or caregiver and child regarding depression and mental health care, T2C provided cues to action at the end of each day in the form of a *talk tip,* for example, “Sometimes starting the conversation is hard. Consider: Are you okay? I’m here if you want to talk.” On day 7, parent or caregiver participants received a check-in asking if they were then ready to make an appointment. At this point, if they did not feel ready, they were referred to their child’s primary care physician to discuss their decisions. If they indicated that they were ready, they received appointment instructions.

#### Adolescent

**Figure 2:**
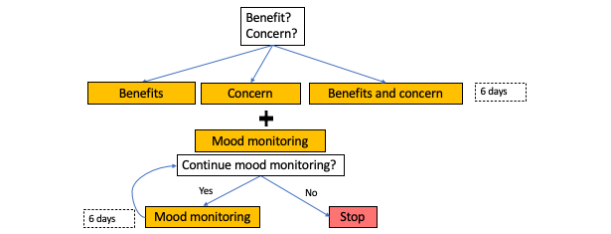
Adolescent Text To Connect flow diagram.

Upon enrollment (Figure 2), adolescent participants received similar introductory queries to their parents or caregivers to help tailor intervention material: “Do you think that seeing a mental health clinician could help you?” and “Do you have any concerns about seeing a mental health clinician?” On the basis of their responses to these queries, an adolescent would receive psychoeducation in the form of *true* or *false* questions for 6 days aimed at reducing stigma, increasing perceived benefits of mental health care, or both (see Multimedia Appendix 1 for sample messages). To increase awareness of symptoms and severity of depression among adolescents, T2C incorporated daily mood monitoring, which has been shown to differentiate adolescents at higher risk for psychopathology [16]; “How often have you felt down, depressed or sad today on a scale 1 (not at all) to 5 (all day)?” As depressed adolescents often underestimate the severity of their symptoms and do not correctly perceive their degree of psychological risk associated with depressive symptoms [17], T2C provided adolescents with both immediate and end-of-week feedback on their depression-related symptoms. Immediate feedback focused on relating symptoms to underlying psychopathology, for example, “You report some symptoms of depression today” and prompting self-management strategies, for example, “Try to focus on one small thing that was good today.” End-of-week feedback focused on quantifying the extent of depressive symptoms; “You reported some symptoms of depression on [X%] of days this past week,” where X was calculated as: (days with any depressive symptoms) / (days with any report). Adolescents were then offered the option of continuing mood monitoring: “Are you interested in continuing to monitor your mood for another week?”

#### Safety

We included a safety feature whereby any response from adolescents or parents or caregivers that included a concerning keyword (ie, help, suicide, kill, hurt, die, pills, gun, and please) immediately triggered an automated message providing crisis resources: “If you are experiencing an emergency, please call 9-1-1 or text CONNECT to 741741 to reach the Crisis Text Line.”

### Phase 1: Focus Groups

#### Study Design

Phase 1 was a focus group study of adolescent-parent or caregiver dyads. All participants completed written informed consent. All procedures were approved by the institutional review board of the University of Pittsburgh.

#### Participants

We recruited **a**dolescent-parent or caregiver dyads from primary care clinics and through flyers posted in clinical settings at a single health system in western Pennsylvania. Research assistants approached families or contacted them by phone after they expressed interest in participating in the research. Eligible adolescent-parent or caregiver dyads were included if the child was aged between 12 and 17 years and screened positive for depressive symptoms as part of standard clinical care at their pediatric primary care office. Participants were not eligible if they were not fluent in English. Participants were scheduled for a focus group session where signed consent was obtained from young people aged >16 years and signed parent or caregiver consent and child assent from those aged <16 years.

#### Procedures

Before conducting any focus groups, a standardized, semistructured qualitative guide was developed to increase consistency across the interview sessions. Data collection for adolescents and parents or caregivers occurred between November 2018 and January 2019. The focus groups took place at research staff offices, and the sessions lasted approximately 120 minutes. Adolescent focus group sessions occurred separately from the parent or caregiver sessions. The focus groups were conducted by a female facilitator with expertise in focus group techniques and qualitative methodology. All sessions were audio recorded digitally and transcribed. A slide presentation of a T2C mockup was shown to the participants as part of the session. The interview topic guide covered (1) barriers to mental health care, (2) ways to motivate teens to initiate mental health care, (3) reactions to the T2C prototype, and (4) design areas to develop further regarding the design and content of T2C. All participants received US $25 for focus group participation.

#### Analyses

Focus group transcripts were analyzed using a thematic content analysis approach and used the qualitative research software package ATLAS.ti 5.0 (Scientific Software Development). A preliminary codebook was created based on close readings of the first transcripts, incorporating explicit domains from interview guides (deductive themes) as well as recurrent unanticipated themes that emerged across transcripts (inductive themes). The coded text was further reviewed through an iterative process, resulting in refined themes. We did not record which individual participant said which statement or counted how many participants agreed or disagreed with a given statement. In presenting the results, we chose participant quotes that represented both most sentiments within each theme as well as any quote that offered a contrasting opinion within that theme.

### Phase 2: Pilot Single-Arm Trial

#### Study Design

We then conducted an open single-arm trial of T2C, preregistered at clinicaltrials.gov (NCT04560075). Data were collected at baseline and 4-weeks post enrollment. **A**dolescents could enroll without their parents or caregivers enrolling. Participants completed written informed consent. All procedures were approved by the institutional review board of the University of Pittsburgh.

#### Participants

We recruited **a**dolescents from 4 primary care sites (22/43, 51%), 1 specialty clinic (2/43, 5%), and 1 mental health clinic (19/43, 44%) in Pittsburgh, PA. Participating sites were asked to identify and refer adolescents with depression who were referred to mental health care according to their usual clinical practice. Upon identifying potential adolescent participants, the care providers asked if the parent or caregiver was willing to speak with a researcher to learn more about the study and provide assent for their child. Once an adolescent was enrolled, an available and interested parent or caregiver (one per child) was assessed for inclusion and, if verified, were enrolled. Once informed consent was obtained, the research team scheduled baseline assessment and relevant onboarding.

Inclusion criteria were as follows: (1) adolescents aged 12-17 years, (2) English language fluency and literacy sufficient to engage in study assessment and intervention, (3) adolescent Patient Health Questionnaire-9>11 or Patient Health Questionnaire-9 item 9>1, and (4) adolescent referred for mental health services by their primary care provider or other health care provider. Exclusion criteria were as follows: adolescents and parents or caregivers who do not own a cell phone with SMS text message capabilities.

#### Measures

Cognitive barrier classification (ie, perceived benefits or stigma) and program engagement data (ie, negative affect check-ins) were obtained from SMS text messaging logs. Demographics, program usability, barriers to mental health care, and health care use measures were obtained through a secure website at baseline and 4-weeks from adolescents.

#### T2C Usability

T2C usability was measured with questions taken from the Post-Study System Usability Scale [18]. The questions included “Overall, I am satisfied with how easy it is to use Text2Connect,” “The information provided with Text2Connect was clear,” and “I liked interacting with the Text2Connect Program,” all with response options ranging from 1=strongly disagree to 7=strongly agree. The question “I needed to learn a lot of things before I could get going with Text2Connect” was presented with response options ranging from 1=strongly disagree to 5=strongly agree. The question “If a friend were in need of a mental health care, would you recommend Text2Connect to help him or her manage symptoms?” was presented with response options ranging from 1=no, definitely not; 2=no, I don’t think so; 3=yes, I think so; and 4=yes, definitely, reduced in analyses to a dichotomous variable of yes or no.

#### Barriers to Mental Health Care

Barriers to mental health care were measured using a brief version of the Barriers to Adolescents Seeking Help scale, derived from the longer scale developed by Kuhl et al [19]. The abbreviated measure comprised 5 of the original 37 self-report items and specifically targets belief-based barriers to seeking professional mental health help (eg, “A therapist might make me do or say something that I don’t want to” and “If I had a problem and told a therapist, they would not keep it a secret”). Each item was presented with response options ranging from 1=strongly disagree to 6=strongly agree. Higher scores indicate higher belief-based barriers to professional mental health help-seeking. In this study, Cronbach α for the 5-item abbreviated measure Barriers to Adolescents Seeking Help was .66 (for adolescents) and .45 (for parents or caregivers). Because internal consistency was low, all item responses are presented separately, and, for simplicity of presentation, we dichotomized responses into agree (if responded agree or strongly agree) and disagree (if responded slightly disagree, disagree, or disagree strongly).

#### Analysis

We assessed engagement by calculating the response rates to SMS text messages within and between individuals. We assessed usability and perceived utility by examining ratings and qualitative feedback from adolescents and parents or caregivers at the 4-week follow-up. Potential effectiveness was assessed by examining changes in perceived barriers to mental health care in adolescents and parents or caregivers from baseline to 4-weeks. We explored the patient characteristics associated with mental health care initiation at 4-weeks using chi-square tests.

## Results

### Phase 1: Focus Groups

#### Sample Characteristics

A total of 9 dyads of adolescents with depression and their parents or caregivers completed a focus group session. Adolescents were aged between 15 and 17 years; 33% (3/9) were female, 33% (3/9) were Black, and 22% (2/9) were more than one race. Parent or caregiver age ranged from 31 to 61 years; 89% (8/11) were female, 22% (2/9) were Black, and 9% (1/11) were more than one race.

#### Theme 1: Parents or Caregivers and Adolescents Face Multiple Barriers to Receiving Mental Health Care

Parents described playing an active role in scheduling appointments but experienced logistical barriers to successfully navigating the system. A mother stated the following:

You know that’s already like kind of an emotionally overwhelming sort of thing to be facing. Um, and to have like all the logistics piled on top with like it’s so complex and you have to navigate it all by yourself.

Parents described a dearth of mental health professionals specializing in adolescents, a need to find the *right fit* with their child’s personality, and a sense of frustration with the need for multiple mental health care providers. For example, one parent quipped, “One for evaluation, one for talk therapy, one for medication.” Parents and adolescents lamented the difficulty in fitting mental health care into their schedules. One parent even described the frustration with their child being *fired* when they missed an mental health care appointment.

#### Theme 2: Most Parents Report Being Supportive of Mental Health Care, but Adolescents Were Frequently Opposed to or Ambivalent About Seeking Mental Health Care

Common reasons adolescents reported being opposed to mental health care included the following: not believing that mental health treatment was necessary, not believing that mental health treatment is effective, not knowing what to expect from therapy, fearing that the counselor would not respect their privacy, and the concern that getting treatment means something is *wrong* with them. An adolescent bluntly summed this up as follows:

Counseling never helps, it’s useless, it’s a waste of time. I do not wanna go talk to somebody. Um, they do not know anything.

#### Theme 3: Adolescents Suggested Many Ways Mental Health Care Could Be Viewed as More Useful

One suggestion was to explain to an adolescent exactly what mental health care actually entailed. As one teen put it, a program should help them realize that through therapy, “they’ll be a better version of themselves [who] will be more aware of how to deal with their emotions properly rather than doing something they regret.” Another suggestion was to present them with evidence of their depression or negative mood to make treatment seem more necessary. A third suggestion was to help the teen find a therapist with whom they could build rapport. Finally, there was the idea that if all else failed, to incentivize the teen through payment. A parent echoed this idea when she said the following:

I think I used bribery, [Laughter] threats of a privilege loss. Um, I’m trying to remember exactly what I did but I-I can’t um, I mean I told him that he needed to go, like he just, he needed to uh, he needed to give it a shot, go at least once or twice.

#### Theme 4: Most Participants Had a Positive Reaction to T2C, and Many Thought That Using T2C Would Achieve the Goal of Increasing the Number of Teens Who Received Treatment

Texting was perceived as an ideal modality as it is easy, relatable, and simpler than talking on the phone. An adolescent said the following:

I think this is fabulous, um every teenager I see nowadays is on their phone non-stop, and I think that they can relate to some—cuz it’s not a live person too. They’re a little bit more—it’s not a parent, it’s not a live person, so if they are having a feeling, they can say “yes” and not like that fear of uh the adult either standing there or like a parent like, you know. Yeah, so I actually love this a lot.

Parents thought it could be useful to convince reluctant parents that their children needed treatment and would provide parents with the same information as their children so that they could be *on the same page*, and also so that the parents could discuss the information with the child. There seemed to be mixed feelings about interacting with an automated communication system or *bot*; they expressed that they were more likely to disclose *true* feelings but also less likely to feel obligated to engage.

#### Theme 5: Adolescents and Parents Had Several Suggestions on How to Improve T2C

Adolescents felt that it was important to be very clear in onboarding about who would see their texted responses, especially responses related to their mood reports. Several adolescents suggested that messages may sometimes not grab their attention and could get dull over time and recommended using humor and emojis to get and keep the teens’ interest. Most adolescents did not like the *true* or *false* format of delivering psychoeducation, relating that some teens may get upset in being told they were *wrong.* Contrary to adolescent concerns about keeping their reports private, several parents thought that there would be value in having access to their child’s mood reports. A parent stated that shared reports could convince “reluctant parents that their children needed treatment...so that they could be on the same page, and also so that the parents could discuss the information with the child.”

### Phase 2: Pilot Single-Arm Trial

#### Participants

##### Adolescents

Of the total sample of consented adolescent participants (n=43), most were born female (35/43, 81%) and White (33/43, 77%; Table 1).

**Table 1:** Characteristics of phase-2 study participants (N=71).

Characteristics			Adolescent (n=43)		Parent or caregiver (n=28)
Age (years), mean (SD)			16 (2.9)		—^a^
Sex at birth (female), n (%)			35 (81)		25 (89)
**Race, n (%)**					
	White	33 (77)		27 (96)	
	Black or African American	3 (7)		1 (4)	
	Asian	2 (5)		0 (0)	
	More than one	2 (5)		0 (0)	
	Other	3 (7)		0 (0)	
	Hispanic	2 (5)		NR	
Baseline depressive symptoms (PHQ-9^b^), mean (SD)			8.9 (5.5)		NR
Lifetime suicide attempts, mean (SD)			2 (1.5)		NR
**Household income (US $), n (%)**					
	<25,000	NR		3 (11)	
	25,000-50,000	NR		5 (18)	
	50,000-75,000	NR		3 (11)	
	75,000-100,000	NR		5 (18)	
	100,000	NR		12 (43)	

^a^Not available.

^b^PHQ-9: Patient Health Questionnaire-9 item.

##### Parents or Caregivers

A total of 28 parents or caregivers agreed to participate (18/28, 65% of dyads Parents or caregivers were mostly female (25/28, 89%) and White (27/28, 96%). Approximately 43% (12/28) reported living in a household that makes >US $100,000 per year.

#### Mental Health Care and Barrier Classification

##### Adolescents

At baseline, before any intervention exposure, 21% (9/43) of adolescents reported not perceiving benefits to mental health care and 21% (9/43) reported perceived stigma to mental health care.

##### Parent or Caregivers

At baseline, before any intervention exposure, 68% (19/28) of parents or caregivers reported already making a first appointment for mental health care for their child. Of the 9 remaining parents or caregivers, 5 (56%) were ready to make an appointment. Of the 4 remaining parents or caregivers who did not feel ready, 1 (25%) reported not perceiving benefits to mental health care for their child, and 1 (25%) perceived stigma to mental health care. Among the 4 matched adolescents whose parents or caregivers were not ready, 2 (50%) adolescents reported not perceiving benefits to mental health care and 1 (25%) reported perceived stigma to mental health care.

#### Adolescent Negative Affect Check-ins

**Figure 3:**
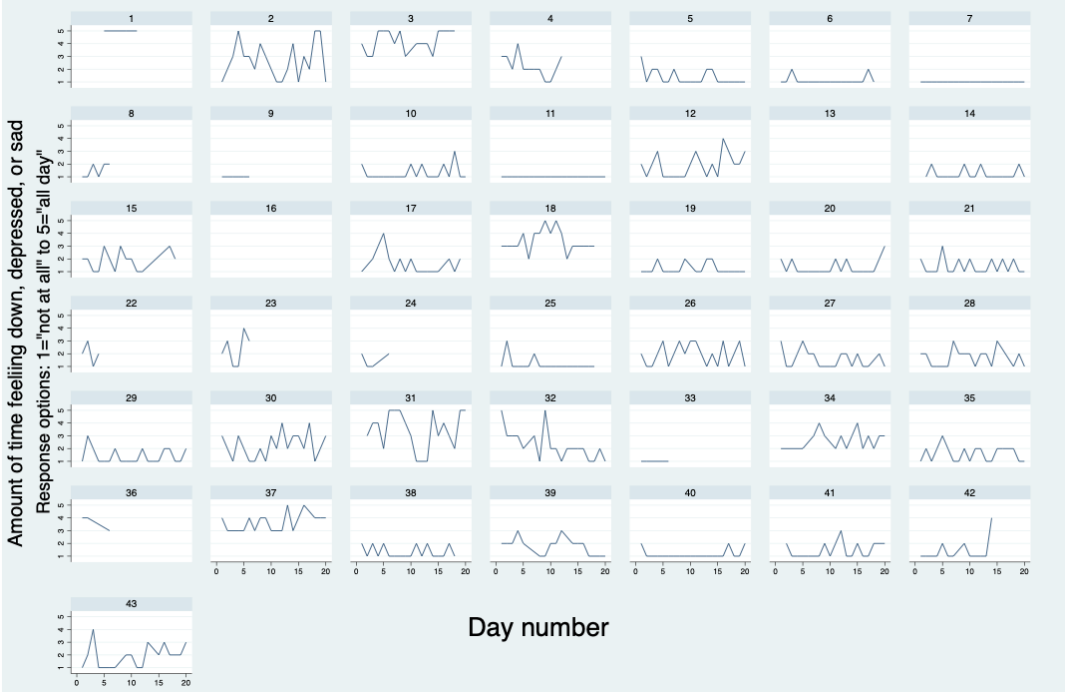
Daily negative affect reports by participant.

A total of 1848 SMS text message negative affect check-ins were sent over the study period and 89.23% (1649/1848) were completed. After completing the first week of T2C engagement, 79% (34/43) of adolescents opted to continue mood monitoring. The median number of weeks an adolescent opted to continue the T2C intervention was 4 weeks and a maximum of 35 weeks in 1 participant. The distribution of responses by adolescent participants to the daily negative affect check-in “How often have you felt down, depressed or sad today on a scale 1 (not at all) to 5 (all day)?” across the first 4 weeks are shown in Figure 3.

#### Program Usability

Overall, 93% (40/43) of adolescents and 96% (27/28) of parents or caregivers completed the usability survey questions (taken from the Post-Study System Usability Scale) at the 4-week follow-up. Overall, mean usability scores were high among adolescents and parents (Table 2).

**Table 2:** Program usability (N=67).

Usability survey questions	Adolescent (n=40)	Parent or caregiver (n=27)
Overall, I am satisfied with how easy it is to use Text2Connect, mean (SD)^a^	6.1 (1.3)	6.5 (1.0)
The information provided with Text2Connect was clear, mean (SD)^a^	6.3 (0.9)	6.3 (1.3)
I liked interacting with the Text2Connect Program, mean (SD)^a^	5.1 (1.5)	5.22 (1.9)
If a friend were in need of a mental health care, would you recommend Text2Connect to help him or her manage symptoms? n (%)^b^	28 (70)	21 (78)
I needed to learn a lot of things before I could get going with Text2Connect, mean (SD)^c^	1.5 (1.0)	1.1 (0.3)

^a^Ratings for statements 1 through 3: 1=strongly disagree to 7=strongly agree.

^b^Ratings for statement 4: 1=no, definitely not; 2=no, I don’t think so; 3=yes, I think so; and 4=yes, definitely was reduced to yes or no and presented as n (%) responding *yes*.

^c^Ratings for statement 5: 1=strongly disagree to 5=strongly agree.

#### Qualitative Feedback on T2C

##### Adolescents

When asked why they would recommend or not recommend the T2C program, adolescents expressed a range of opinions and thoughts. Among those who would recommend the program, several highlighted the benefits of check-ins, noting it made them feel “less alone” and “cared for.” Several others noted the ease of interacting with the program. Among adolescents who would not recommend the T2C program, 1 participant stated, “I think it's good for tracking your mood and how you’re feeling on a regular daily basis but it's not actually helping you cope with your feelings.” Another stated, “I would say it you’re really in a bad mental state having an automated bot is not the best thing to have because it doesn't feel special to you, no real human connection.”

##### Parent or Caregivers

Among those who would recommend the program, parents or caregivers noted the ease of use, 1 parent stating, “It was more laid back for my son than talking to someone in person.” Another commented, “It made you think about the scenarios. easier to open up about things. made recommendations. Text is easy these days and how kids like to communicate.” Several of those who would not recommend the T2C program commented that automation was limiting; one parent or caregiver stated as follows:

Not really interactive. Try to respond to text...we don't understand response call 911 if emergency. Disjointed. If someone was reaching out for help it wouldn't be helpful.

#### Barriers to Mental Health Care

##### Adolescents

**Figure 4:**
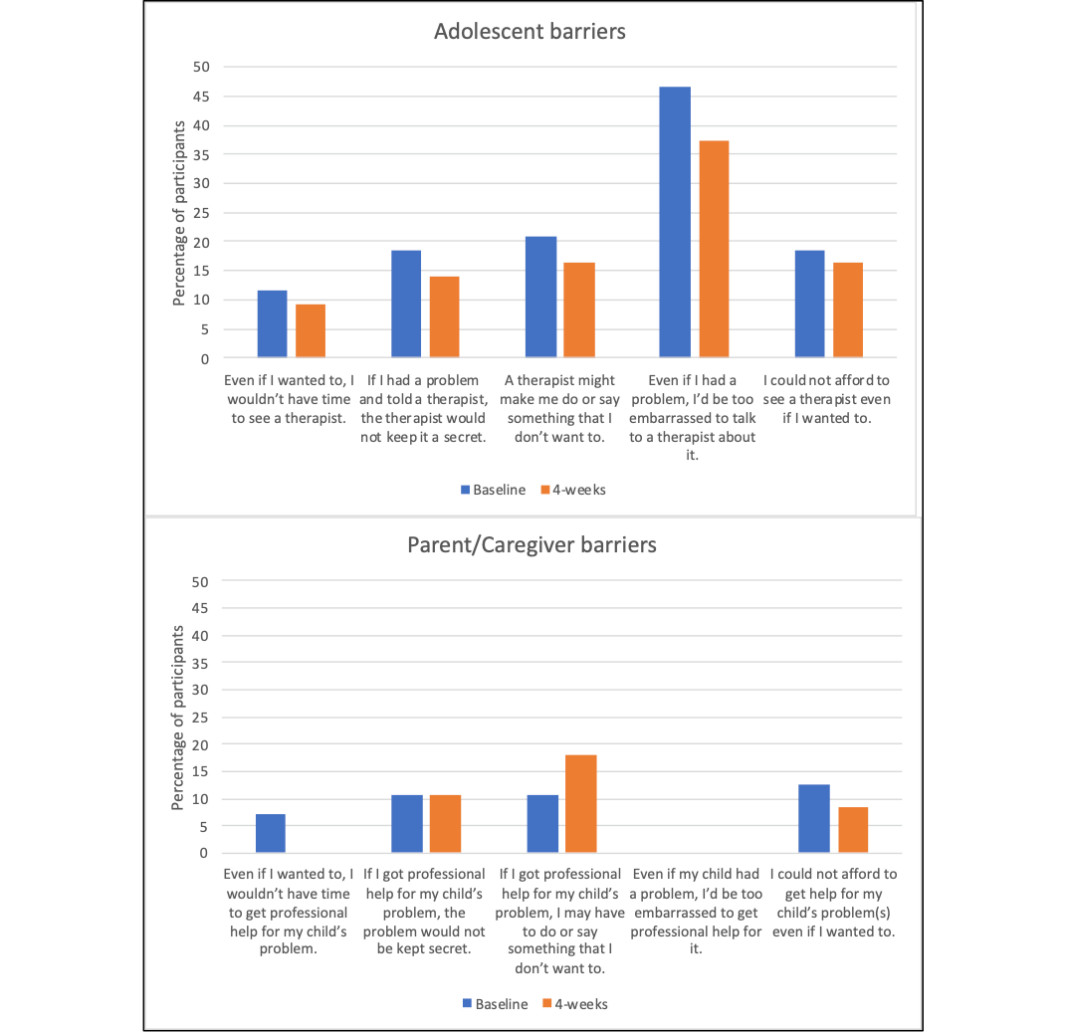
(A) Adolescent barriers and (B) parent or caregiver barriers.

At baseline (Figure 4), embarrassment was the most common barrier to mental health care, and lack of time was the least common barrier. There were small reductions (2%-10%) in all barriers to mental health care between baseline and the 4-week follow-up.

##### Parent or Caregivers

At baseline, barriers to mental health care were less common in parents or caregivers compared with adolescents. Embarrassment was not indicated as a barrier among any parent or caregiver; however, cost was the most common parent- or guardian-reported barrier. There were no reductions in parent- or guardian-reported cognitive barriers to mental health care from baseline to the 4-week follow-up.

#### Mental Health Care

At 4 weeks, of the 42 adolescents who completed follow-up, 22 (52%) reported that they had attended an appointment with an mental health specialist. Of the 20 adolescents who had not attended an mental health appointment, 1 (5%) reported that it was scheduled for a future date, 2 (10%) reported that the primary care site did not have the ability to help them schedule an mental health care appointment, and 3 (15%) reported that they were no longer interested in mental health care.

## Discussion

### Principal Findings

In phase 1, we found evidence that adolescents with depression and their parents or caregivers face numerous barriers to receiving mental health care, among which cognitive barriers related to stigma and belief in the utility of mental health care play a significant role. We also found evidence that a program like T2C incorporating daily negative affect check-ins for adolescents and (for parent or caregivers) daily prompts to talk about mental health care with their child had the potential to reduce some barriers by being easy, relatable, and simpler than talking on the phone. In phase 2, we found evidence that T2C is used at high rates by adolescents, has high usability ratings, and may reduce perceived barriers over time.

Regarding adolescent engagement, we found that all completed onboarding, including mental health barrier classification, and there were high opt-in rates for continued daily negative affect check-ins beyond the first week. For adolescents, we believe that the brevity of daily interactions and the use of SMS text messaging contributed to these high engagement rates. Regarding parent or caregiver engagement, we found that although less than three-quarters of parents consented to enroll in T2C, all who did completed onboarding, including completion of mental health barrier classification. Not surprisingly, for adolescents with parents who refused enrollment, we found lower rates of mental health care initiation at follow-up. Future studies should seek to understand why certain parents or caregivers defer enrollment and ways to improve parental or caregiver enrollment in help-seeking interventions.

Usability ratings and qualitative feedback reinforced engagement findings. Overall, adolescents and parents or caregivers were satisfied with the T2C program, found the message content clear, and did not need to learn things before operation. In addition, around three-quarters of adolescents and parents or caregivers would recommend the T2C program to someone in need. From qualitative feedback, in addition to being seen as easy to use, the T2C program—especially the daily negative affect check-ins—seemed to provide some feeling of being cared for. The limitation of automated programs not being able to provide truly personalized support was brought up, suggesting that efficient programs that supplement *bot* with counselor communications should be considered [20].

At the 4-week follow-up, most adolescents reported either having initiated mental health care or pending scheduling, leaving only 15% (3/20) of adolescents still not interested in mental health care. On the one hand, this seems to be a higher rate of mental health care initiation than the 30% reported in the literature. On the other hand, this indicates that additional program components or strategies are needed to close this gap. At baseline, almost three-quarters of parents or caregivers reported already making an mental health care appointment, and more than half of those remaining were ready to make an appointment. For these dyads, the T2C program functioned essentially as a scheduling assistant. For parents or caregivers who were not ready, the program sought to reduce cognitive barriers and promote dialog with their child about mental health care. Additional features may include strategies to overcome structural barriers to mental health help-seeking, including transportation and virtual visits.

Mechanistically, we found some evidence that the T2C program facilitates reductions in adolescent cognitive barriers to mental health care from baseline to 4 weeks. The greatest reduction (47% to 37%) was observed for concerns around embarrassment, with smaller reductions regarding privacy and disclosure. We speculate that the daily negative affect check-ins with feedback may have made adolescents more comfortable sharing mental health symptoms, which in turn helped reduce concerns about embarrassment. Future intervention design could consider enhancing this effect by priming the adolescent-provider relationship before an in-person visit with asynchronous communication.

### Limitations and Strengths

This study has several limitations. First, we recruited mostly female White youth; therefore, the findings may not be valid in males or racial or ethnic minorities. Second, we did not measure objective mental health care initiation but used self-reports from adolescents. Third, we did not capture participants’ specific mental health or substance use diagnoses in addition to depression, limiting the understanding of severity and breadth of mental health care needs. We note several strengths of our study. First, we were able to recruit youth and parent or caregiver dyads, as both are critical in understanding barriers to mental health care initiation. Second, we designed and evaluated the intervention through multistage user feedback, incorporating mixed methods. Third, in the phase-2 open trial, we examined multiple sources of outcome data within each participant to *triangulate* usability findings.

### Conclusions

In conclusion, this two-phase study demonstrates the need to engage both adolescents and parents or caregivers in interventions aimed at overcoming barriers to mental health care initiation and the potential utility of digital strategies focused on cognitive and motivational barriers to help-seeking to do so. There is an urgent need for evidence-based help-seeking programs for youth with depression, and a program like T2C, if found to be effective in a larger trial, could fill a needed gap.

## Appendix

Multimedia Appendix 1 Sample messaging.

## Abbreviations

T2C: Text To Connect
