# Supplementary material for: A Text Message Intervention for Adolescents With Depression and Their Parents or Caregivers to Overcome Cognitive Barriers to Mental Health Treatment Initiation: Focus Groups and Pilot Trial
Source: JMIR Form Res. 2021 Nov 9;5(11):e30580. doi: 10.2196/30580 (PMC8663469; doi:10.2196/30580)
Supplement: Multimedia Appendix 1 [file formative_v5i11e30580_app1.docx]

**Benefits Psychoeducation**

| **TRUE or FALSE: Most kids experience low moods most days** |
| --- |
| Although most kids have occasional low moods, when it occurs most days it may be a sign of depression |
| Correct: Although most kids have occasional low moods, when it occurs most days it may be a sign of depression |
|  |
| **TRUE or FALSE: Kids with depression can snap out of it if they want to** |
| Typically, depression can’t be cured by thinking positively. A mental health provider can provide the tools needed to help |
| Correct. It typically can’t be cured by thinking positively. A mental health provider can provide the tools needed to help |
|  |
| **TRUE or FALSE: Kids with depression just need to resume their normal routines to get better** |
| It is difficult for kids with depression to do this by themselves. A mental health provider can help |
| Correct. It is difficult for kids with depression to do this by themselves. A mental health provider can help |
|  |
| **TRUE or FALSE: Kids with depression need to be in treatment for their whole lives** |
| The length of mental health care for depression varies widely. Most youth do not need to be in treatment for their lives |
| Correct. Most youth do not need to be in treatment for their lives |
|  |
| **TRUE or FALSE: People with depression don’t get better** |
| A mental health provider can give people with depression the best chance at getting better |
| Correct. A mental health provider can give people with depression the best chance at getting better |
|  |
| **TRUE or FALSE: Up to 90% of adolescents with depression will get better with mental health care** |
| Correct! Mental health care for depression is highly effective... up to 90% of people feel better |
| Actually, mental health care for depression is highly effective... up to 90% of people feel better |

**Concern Psychoeducation**

| **TRUE or FALSE: Only people with severe depression go to a mental health clinician** |
| --- |
| Even people with mild symptoms can benefit from seeing a mental health clinician |
| Correct. Even people with mild symptoms can benefit from seeing a mental health clinician |
|  |
| **TRUE or FALSE: More than 1 in 5 teens has a mental health condition like depression or anxiety** |
| Correct. More than 1 in 5 teens has a mental health condition. |
| More than 1 in 5 teens has a mental health condition. |
|  |
| **TRUE or FALSE: Seeking mental health care means there is something "wrong" with me** |
| Actually, seeking help for mental health is a sign of a strong individual who is able to recognize their needs. |
| Correct. Seeking help for mental health is a sign of a strong individual who is able to recognize their needs. |
|  |
| **TRUE or FALSE: A mental health care provider won't understand my problems** |
| They may not always understand exactly how you are feeling, but they will use their experience to try to help you. |
| They may not always understand exactly how you are feeling, but they will use their experience to try to help you. |
|  |
| **TRUE or FALSE: Mental health clinicians will make me share private things** |
| When you see a mental health provider, you do not have to talk about anything you don't feel comfortable with |
| Correct. When you see a mental health provider, you do not have to talk about anything you don't feel comfortable with |
|  |
| **TRUE or FALSE Talking about how bad you feel will only make things worse** |
| It can feel uncomfortable to talk about your feelings at first. But many people find it helps to talk to a mental health provider |
| Correct. It can feel uncomfortable to talk about your feelings at first. But many people find it helps to talk to a mental health provider |

**Negative Affect Replies**

| **Incoming Report** | **Outgoing Message** |
| --- | --- |
| 1 | We are sorry to hear that. Let someone you trust know how you feel |
| 2 | Try to focus on one small thing that was good today |
| 3 | A mental health clinician could help give you the tools to manage these feelings |
| 4 | try: https://www.nimh.nih.gov/health/publications/teen-depression/index.shtml |
| 5 | Getting active can help you to feel better. Try taking a walk, a bike ride |
| 6 | If at any time you feel you need help immediately, text “safe” to 4HELP (44357) |
| 7 | To help you lift your mood, try listening to a favorite song, painting, or playing an instrument |
| 8 | Consider writing down in a journal a good memory you've had |
